# Health literacy in a high income Arab country: A nation-wide cross-sectional survey study

**DOI:** 10.1371/journal.pone.0275579

**Published:** 2022-10-05

**Authors:** Satish Chandrasekhar Nair, Jayadevan Sreedharan, Karthyayani Priya Satish, Halah Ibrahim

**Affiliations:** 1 Department of Academic Affairs, Tawam Hospital, Al Ain, United Arab Emirates; 2 College of Medicine, UAE University, Al Ain, United Arab Emirates; 3 Department of Community Medicine, College of Medicine, Gulf Medical University, Ajman, United Arab Emirates; 4 Kasturba Medical College, Medicine Program, Mangalore, Karnataka, India; 5 Khalifa University College of Medicine and Health Sciences, Abu Dhabi, United Arab Emirates; Taipei Medical University, TAIWAN

## Abstract

**Introduction:**

Health literacy is a powerful predictor of health outcomes, but remains a global challenge. There is a paucity of published data and limited understanding of the health literacy of patients in the Middle East. The purpose of this study was to assess the patient health literacy levels in the United Arab Emirates (UAE) and identify associated demographic characteristics.

**Methods:**

A cross-sectional survey of adult patients attending public and private hospitals and primary care clinics was conducted across the UAE between January 2019 and May 2020. Chi-square test was used to analyze the association between health literacy and demographic variables. Ordinal regression was adopted to analyze the data for statistically significant independent variables.

**Results:**

2349 of 2971 patients responded (79% response rate). Slightly less than one-quarter (23.9%) of patients surveyed demonstrated adequate health literacy. Over a third of women respondents (31.7%) possessed adequate health literacy, as compared to only 13% of men surveyed (p<0.001). Participant age was significantly (p<0.001) associated with health literacy levels, with approximately 50% of participants above age 50 years (51–75 years) demonstrating inadequate health literacy. Education was also positively correlated with health literacy. Adequate health literacy levels were twofold higher (30.5%, p<0.001) in patients with high school education, as compared to patients without secondary education.

**Conclusions:**

The high proportion of patients with inadequate health literacy in our study confirms that the health literacy deficit is a challenge in the UAE. Targeted interventions are needed to improve health literacy, particularly for older individuals, to optimize healthcare utilization and improve individual and population health outcomes.

## Introduction

Health literacy is a powerful predictor of health outcomes. According to the World Health Organization (WHO), it is one of the most important health indicators [1, 2]. Health literacy goes beyond the ability to read prescription labels or complete patient information forms; it encompasses all of the skills necessary to obtain, understand and process basic health information and services in order to make important health decisions [3]. Numerous studies have documented an association between health literacy and health outcomes [4, 5]. For individuals, low health literacy is associated with decreased use of preventive and screening services, decreased compliance with medications and treatment plans, increased use of emergency services, and increased hospitalization. Populations with low health literacy experience inefficient usage of healthcare services, increased healthcare costs and, ultimately, higher mortality rates [4, 5].

Health literacy is a global public health concern. Studies in multiple countries have documented overall low health literacy levels [6]. A large-scale survey in the United States found that only 12% of Americans demonstrated an adequate level of health literacy, whereas 36% had basic and below basic health literacy [7]. Similarly, the European Health Literacy survey revealed that 47% of the respondents demonstrated limited health literacy, 12% displayed inadequate health literacy, and an additional 35% had problematic health literacy [1]. Despite recognition of the impact of health literacy on individual and population health, considerable gaps in the literature exist regarding health literacy levels in many countries. There is a paucity of published data and limited understanding of the health literacy of patients in the Middle East, with a recent review showing that health literacy research has only been conducted in ten Middle Eastern countries, and mostly in Iran, Saudi Arabia and Turkey [8, 9]. Identifying health literacy levels and implementing measures to improve health literacy can lead to improved patient and societal health outcomes.

Over the past two decades, many countries in the Middle East, particularly in the Gulf Cooperation Council (GCC) countries of Kuwait, Qatar, Oman, Saudi Arabia, Bahrain and the United Arab Emirates (UAE), have witnessed substantial demographic and economic growth [10]. The UAE is a small Middle Eastern country with a population of over 9 million residents. Supported by its control over a large petroleum supply, the UAE has transformed from a primitive Bedouin society into a major business and political global force in just under fifty years. In line with its Vision 2030 initiative and the United Nation’s Sustainable Development Goals [11], the UAE has made considerable investments in education and healthcare infrastructure, with state-of-the-art medical equipment and facilities [10]. Yet, this rapid development and urbanization has led to untoward consequences, including increased rates of obesity and lifestyle related diseases, namely diabetes, hypertension and cardiovascular disease [12]. It has been well documented that patients with chronic diseases, such as diabetes, have low health literacy [13], suggesting that patients with the greatest need for health information often have the least ability to access it. Further, the UAE’s multicultural and multilingual patient population is cared for by a high number of expatriate health professionals [14]. The increase in chronic diseases, along with language and cultural barriers within the modern and often complex medical system of the UAE, can create obstacles to healthcare access and utilization and, therefore, warrant investigation into the role of health literacy in disease outcomes. Through a nationwide health literacy survey, we sought to answer the following questions:

1. What is the overall health literacy level of the multicultural, multiethnic patient population in the UAE?
2. Which demographic characteristics are associated with health literacy?

## Methods

### Study design and setting

A nationwide cross-sectional survey was conducted across all seven emirates in the UAE between January 2019 and May 2020. Participants were recruited from outpatient waiting areas in public and private hospitals and primary healthcare clinics in the UAE by multilingual physicians blinded to the study objectives. Inclusion criteria mandated that participants be 18 years of age or older, as confirmed by their national identification card, residents or citizens of the UAE, able to understand and answer the survey questions, and agree to provide written informed consent. Patients were recruited from several different specialty clinics, namely cardiology, dental, infectious diseases, metabolic diseases, and surgery. Following the informed consent process, physician researchers conducted face-to-face interviews with the participants and electronically recorded their responses using an iPad. Visuals of the local and English language newspapers, currency denominations, and the various hospital signs relevant to the survey items were displayed on the iPad.

### Ethical approval and informed consent

Ethical approval for a multi-center study was obtained from the Al Ain Medical District Human Research Ethics Committee (AAMDHREC 13/55 & REC 01.10.2013RS279) and the Tawam Hospital Research Ethics Committee (702–2019). Approval for data collection was also obtained from the senior management of each participating hospital and clinic. Written informed consents were obtained from the study participants. In patients with low literacy, verbal agreement was obtained prior to written consent, and when available, a family member also provided written informed consent. Medical records were not accessed at any site and no personal health information was obtained. A computer-assigned random study number was assigned to each participant after obtaining informed consent so that no personal identifying information was collected. Informed consent, participant privacy and patient data confidentiality were strictly adhered to in accordance with the International Conference on Harmonization-Good Clinical Practice guidelines, and all the methods conformed to the principles embodied in the Declaration of Helsinki.

### Survey instrument

The Eastern-Middle Eastern-Adult-Health Literacy 13 questionnaire (EMAHL13) was used for this study because it was developed in the UAE and showed high validity evidence in the UAE population [15]. It is also brief, simple to administer, and available in the most prevalent languages in the UAE, namely Arabic, Hindi, Urdu, Tagalog, and Malayalam. The design, development and validation of the EMAHL13 have been previously reported [15]. The survey is comprised of 13 items categorized into 4 domains, representing different activities in which patients engage with their healthcare system, namely 1) completing medical forms, 2) reading patient information materials, 3) navigating the health care system, and 4) differentiating medications. A five-point Likert scale was used to assess participant responses “1 = never, 2 = rarely, 3 = sometimes, 4 = most of the time, and 5 = always.” The mean score for all item responses ranged from a minimum of 13 to a maximum of 65 for each participant, with 1–26 (never/rarely) signifying inadequate health literacy, 27–39 (sometimes) indicating marginal health literacy, and 40–65 (most of the time/always) representing adequate health literacy. Adequate health literacy was defined as the ability to obtain, process, and understand basic health information in order to make appropriate health decisions [1].

### Sampling method & sample size

To our knowledge, there are no published studies of health literacy in the UAE. An estimate of health literacy prevalence for the population of the Gulf countries was derived from prior national studies in the region, in which the average adequate health literacy prevalence for all ages was approximately 20% [16, 17]. Therefore, the minimum sample size representative of the population required was estimated to 1850 participants. Previous experience indicated a 20% loss in the completed survey return rate, which was compensated [17]. We adopted a purposive sampling method to collect a minimum of 2220 completed surveys.

### Data analysis

Based on a review of the literature, factors contributing to patient understanding of health literacy were determined for the study, specifically patient age, gender, education levels, and nationality. Categorical variables included gender, age (categorized into 18–30, 31–50, 51–65, and 66-75years) education (categorized into no secondary education, high school, and bachelors and post graduate degree), and nationality (country of origin categorized as GCC, Arab world (non-GCC), Asia, Europe and North America, and Africa).

Data were analyzed using SPSS Statistical Software Version 27 (SPSS Inc. Chicago, USA). The association of the dependent variable (health literacy level) with other independent variables was conducted using the Chi-Square test. To adjust for confounders, we used ordinal regression since the dependent variable is ordinal (inadequate, marginal, adequate). All independent variables, which showed statistical significance in Chi-square test, were included in the ordinal regression. We used the Strengthening the Reporting of Observational studies in Epidemiology (STROBE) checklist for cross-sectional studies when writing our report [18].

## Results

**Fig 1:**
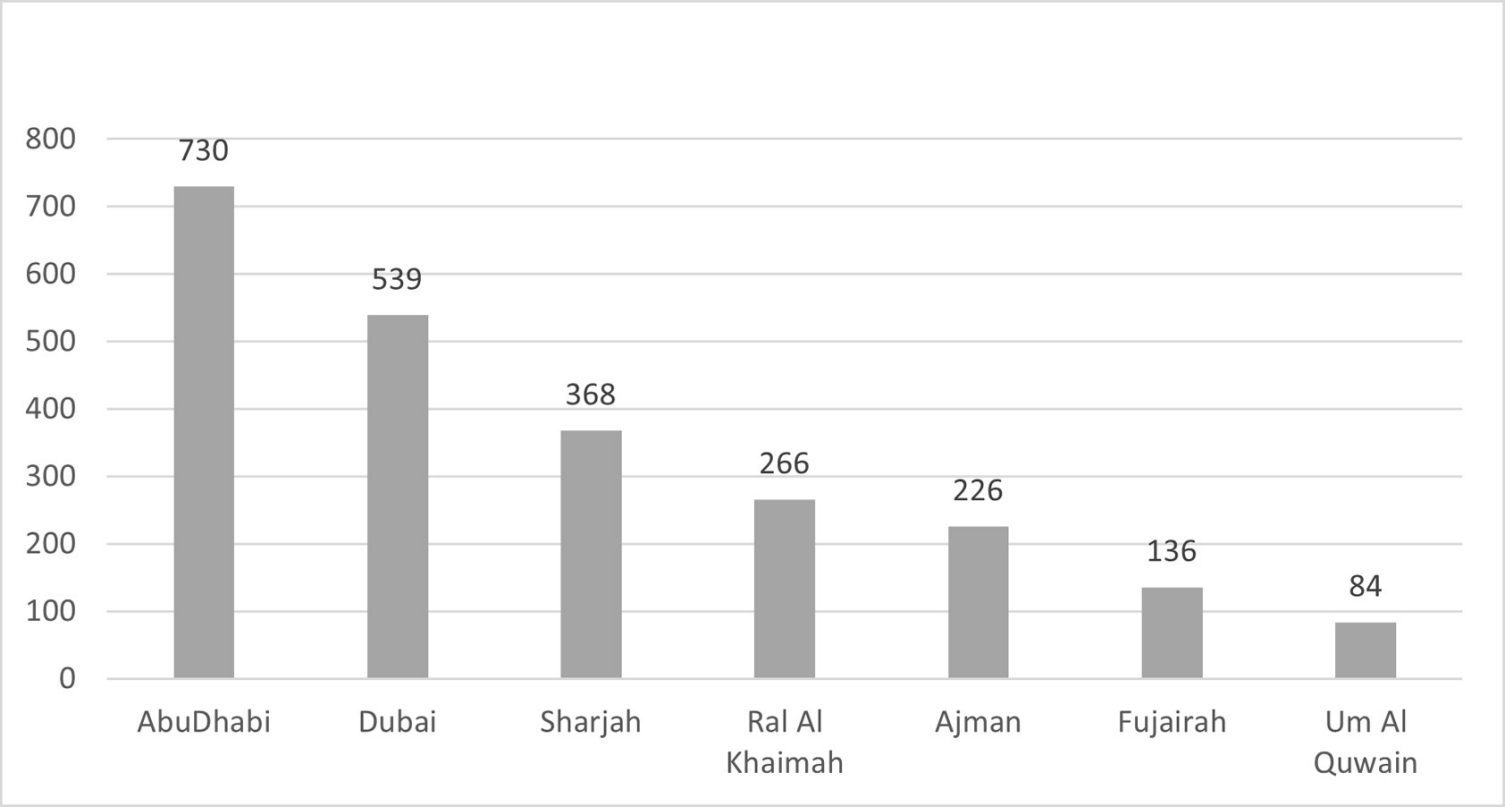
Participant distribution across the seven emirates of the United Arab Emirates (N = 2349).

From a total of 2971 patients, 622 individuals did not meet the inclusion criteria or refused to provide written informed consent and were excluded from the study. The majority of the excluded patient participants (377/622, 59.4%) did not have their original national identification card with them, and it was not possible to verify their age, nationality or residency status. We excluded individuals above the age of 75 because there was an insufficient number of participants in this age group (29/622, 4.6%). The remainder of excluded individuals (216/622, 34.7%) did not provide informed consent. A total of 2349 patients (79.1%) were included in data analysis. Thirty-one percent (730) of the total patient participants were from Abu Dhabi, the largest emirate and capital of the UAE, followed by 22% (539) from Dubai, the second largest emirate. The lowest patient participation (3.5%) was from Um Al Quwain (Fig 1).

Participant demographics are listed in Table 1. The majority of the survey participants were women (1369/2349, 58.3%), and 44.7% (1050/2349) were from the GCC countries. The median age of the population for the current study is 37 years, representative of the median age of the UAE population, which is 33 years [19]. More than half of the participants (1450/2349, 61.7%) were in the 31–65 year age group. The majority (1700/2349, 72.3%) attained at minimum a high school education. Overall, 23.9% of surveyed patients demonstrated adequate health literacy.

**Table 1:** Demographics of participants in the UAE outpatient clinics (N = 2349).

Demographic Variable	n (%N)
**Gender**	
Men	980 (41.7)
Women	1369 (58.3)
**Age (years)**	
18–30	398 (16.9)
31–50	646 (27.5)
51–65	804 (34.2)
66–75	501 (21.3)
**Education**	
No secondary education	629 (27)
High School	1023 (43.6)
Bachelors/Postgraduate degree	677 (29.1)
**Nationality**	
GCC	1050 (44.6)
Arab (not GCC)	434 (33.6)
Asia	595 (69.2)
Europe/North America	181 (68.5)
Africa	83 (3.5)
**Clinics**	
Cardiology	336 (14.3)
Dental	286 (12.2)
Infectious Diseases	526 (22.4)
Metabolic	640 (27.2)
Surgery	561 (23.9)

GCC = Gulf Cooperation Council.

Association analysis (Table 2) between health literacy levels and demographics revealed that women demonstrated almost 2.5 fold (428/1369, 31.3%, p<0.001) higher adequate health literacy, as compared to male survey respondents (127/980, 13%). Yet, a significantly higher percentage (702/1369, 51.3%) of the female participants possessed inadequate health literacy. Participant age was significantly (p<0.001) associated with health literacy levels. Sixty three percent of younger participants aged 18–30 years of age (252/398; 63.3%) possessed adequate health literacy. Approximately 50% of the patient participants above age 50 years (51–75 years) demonstrated inadequate health literacy. Education was also positively correlated with health literacy. Adequate health literacy levels were twofold higher (30.5%, p<0.001) in patients with high school education, as compared to patients without secondary education. Holding a bachelors or post-graduate degree did not improve adequate health literacy levels, when compared to individuals with high school degrees. Patient participants from the GCC countries possessed significantly higher adequate literacy levels (27.5%, p<0.001), followed by patients from Asia (23.7%). Less than 20% adequate health literacy was noted among participants from Africa (13.3%), the non-GCC Arab world (17.5%), and America and Europe (18.8%).

**Table 2:** Association between health literacy levels and participant demographics (N = 2349).

Variable	Group	Literacy Levels			p Value
		Inadequate	Marginal	Adequate	
		n (%)	n (%)	n (%)	
**Gender**	Male	136 (13.9)	717 (73.2)	127 (13.0)	<0.001
	Female	702 (51.3)	239 (17.5)	428 (31.3)	
**Age Group**	18 - 30Y	38 (9.5)	108 (27.1)	252 (63.3)	<0.001
	31 - 50Y	156 (24.1)	248 (38.4)	242 (37.5)	
	51 - 65Y	394 (49.0)	355 (44.2)	55 (6.8)	
	66-75Y	250 (49.9)	245 (48.9)	6 (1.2)	
**Education**	No Secondary Education	279 (44.4)	258 (41.0)	92 (14.6)	<0.001
	High School	271 (26.5)	440 (43.0)	312 (30.5)	
	Bachelors/ Post Graduate Degree	273 (40.3)	253 (37.4)	151 (22.3)	
**Nationality**	GCC	366 (34.9)	391 (37.2)	293 (27.9)	<0.001
	Arab (non GCC)	178 (41.0)	180 (41.5)	76 (17.5)	
	Asia	151 (25.4)	303 (50.9)	141 (23.7)	
	Europe/North America	107 (59.1)	40 (22.1)	34 (18.8)	
	Africa	32 (38.6)	40 (48.2)	11 (13.3)	

In order to determine health literacy levels within the patient populations, ordinal logistic regression models were employed. After analyzing the regression models by taking into account factors with p < 0.05, Table 3 was computed using four independent variables–gender, age, education levels and nationality. The Nagelkerke Pseudo R square for the model was 0.33, indicating that 33% of the dependent variable (health literacy) variation could be predicted by the independent variables (gender, age, education, nationality) included in the model. The model containing all predictors was statistically significant, except for age and education at one level each (Table 3). This demonstrated that the model was able to distinguish between respondents with inadequate, marginal and adequate levels of health literacy. Overall health literacy levels were higher among participants from Asia. Similarly, men possessed higher overall health literacy than women. Also, participants younger than age 50 possessed higher overall health literacy when compared to older patients. To assess the model’s goodness-of-fit, Pearson Chi-square test and deviance were used. Chi-square statistics showed a level of significance of p <0.001; deviance with level of significance p<0.001 suggesting a good fit of the model on the available data.

**Table 3:** Ordinal regression model-determination of marginal and adequate health literacy.

		Estimate	Stand. Error	Wald	df	Sig.	95% Confidence Interval		OR
							Lower Bound	Upper Bound	
Threshold	[Health literacy = 1.00]	-0.23	0.21	1.25	1	NS	-0.64	0.18	
	[Health literacy = 2.00]	2.14	0.21	100	1	< .001	1.72	2.56	
Location	[Gender = Male]	0.69	0.09	63.84	1	< .001	0.52	0.86	2
	[Gender = Female]	0^a^	.	.	0	.	.	.	1
	[Age = 18-30Y]	3.62	0.21	310.66	1	< .001	3.22	4.03	37.4
	[Age = 31-50Y]	1.38	0.19	53.45	1	< .001	1.01	1.75	4
	[Age = 51-65Y]	-0.18	0.19	0.87	1	NS	-0.54	0.19	0.8
	[Age = 65-75Y]	0^a^	.	.	0	.	.	.	2.7
	[Education = No Secondary Education]	0.73	0.19	15.36	1	< .001	0.37	1.1	2.1
	[Education = High School	0.18	0.11	3.03	1	NS	-0.02	0.39	1.2
	[Education = Bachelor’s & Post Graduate Degree	0^a^	.	.	0	.	.	.	1
	[Nation = GCC	-1.45	0.17	72.24	1	< .001	-1.79	-1.12	0.2
	[Nation = Middle East Arabs]	-0.6	0.12	23.47	1	< .001	-0.85	-0.36	0.5
	[Nation = Asia]	0^a^	.	.	0	.	.	.	1
	[Nation = America and Europe]	-0.93	0.17	28.87	1	< .001	-1.27	-0.59	0.4
	[Nation = Africa]	-1.1	0.24	21.95	1	< .001	-1.56	-0.64	0.3

NS = not significant.

## Discussion

The provision of high quality, patient-centered care requires patients to be active participants in their medical management [20]. Inadequate health literacy can be a major obstacle to patient engagement in care and compliance with treatment plans [4]. Consistent with studies conducted in the West, our study in the UAE revealed that only 23.9% of surveyed patients demonstrated adequate health literacy. As there is limited data on health literacy in the Middle East, this first UAE-wide study adds to the health literacy literature. Our results also support a recent cross-sectional national survey of over 3500 individuals in Saudi Arabia, which noted that 46% of respondents had inadequate health literacy [21].

### Demographic characteristics associated with health literacy

Our findings also identify vulnerable groups with higher amounts of low health literacy, particularly older adults. It is notable that individuals above the age of 50, who often have medical comorbidities and disproportionately utilize healthcare resources [22, 23], have significantly lower levels of health literacy. Other studies have also found lower health literacy levels in older patients [20, 22]. These findings are concerning as studies have shown that older adults with inadequate health literacy levels express greater dissatisfaction with their physicians and the healthcare system in general [23]. Targeted interventions are necessary to prevent the marginalization of this patient population and to ensure that older patients are fully engaged with all healthcare decisions.

Adequate health literacy levels were significantly higher among women in our study. Higher health literacy skills in women have also been documented in studies in other countries, including Korea and the United States [22, 24]. Research suggests that women have higher healthcare utilization and are, thereby, more experienced in navigating the healthcare system, which may explain the gender health literacy gap [25]. In addition, traditional gender roles in the UAE more often place women in the caregiver role, caring for sick family members and children. This increased interaction and familiarity with the healthcare system may also contribute to the higher adequate health literacy levels in the women respondents. The overall higher health literacy level in men is an important area for future research.

Our findings confirm that low health literacy and low education levels are closely correlated [26]. Studies focusing on health disparities have consistently identified a strong association between poor health outcomes and lower levels of education [26]. Health literacy is believed to be the underlying mechanism driving this relationship [26, 27]. Therefore, strategies to improve health literacy may also be helpful in reducing health disparities in individuals with lower educational attainment. Our results showed that participants with a college or postgraduate degree did not have higher adequate health literacy levels than individuals with high school diplomas. This finding suggests that although low education levels may correlate with low health literacy, this relationship may not be linear throughout the continuum of education and as higher education levels are achieved. More studies on educational attainment and health literacy are needed.

As the UAE and other Gulf countries continue to invest in the healthcare sector, the assessment of health literacy levels can direct the redesign of healthcare services. Our findings have many policy implications. A longitudinal and multilevel health literacy strategy is needed for the UAE to support the prevention and control of communicable and non-communicable diseases and to improve population health outcomes. First, in light of the large numbers of UAE outpatients with low health literacy levels, clinics must allocate appropriate time for appointments to ensure that patients have adequate time to have all of their questions addressed. Also, patient-centered communication protocols can be developed and implemented throughout the country’s hospitals and clinics. Healthcare professionals should recognize and assess every patient’s ability to understand and process the healthcare information that is provided. This is especially important in older patients and when new technologies, such as web-based modalities or telehealth services, are introduced [22, 28]. Continuing professional development programs, focused on optimizing patient-clinician communication skills, are needed to help physicians develop and improve skills for effective interactions with low health literacy patients. Health professionals should routinely integrate universal health literacy precautions during all patient interactions. These include explaining information in small, easily understandable pieces, frequently checking for comprehension, and using simple language instead of medical terminology. For example, Teach-Back- asking patients to explain in their own words what they have learned- is a best practice that health professionals can use to assess patient understanding [29]. The use of visual aids and pictures has also been shown to improve patient understanding [30]. Further, several large tertiary care centers in the UAE have employed multilingual case managers, social workers and patient advocates working directly with the clinical team to explain health services in detail to hospitalized patients. Expanding these supportive services to primary care services and outpatient clinics may help to increase health literacy, decrease health disparities, and ultimately improve patient outcomes.

In addition to clinician communication training, programs are needed to empower patients and improve their health literacy awareness and skills. Studies using different educational modalities, including formal classes, home visits, and online interventions, have all shown success [31]. For example, a six-month educational intervention for older adults, which included weekly active learning sessions that promoted healthy lifestyle behaviors, was effective in improving health literacy, memory and verbal fluency, balance, physical activity, and dietary variety [20].

Additional targeted interventions are needed for older individuals and those with chronic illness. Chronic diseases account for the majority of health problems in the UAE [12], and have numerous social, psychological, physical, and economic consequences. Management of chronic diseases often requires extensive self-care education and management. Studies have documented overall low health literacy levels in individuals with chronic illness [32]. As such, programs to improve health literacy in this group could ultimately improve population health outcomes. Finally, a modifiable factor that may enhance health literacy is education, thus stressing the need to enhance literacy to scale-up health literacy.

Our study spanned a large age range and multiple health conditions. Our findings must, however, be viewed in light of some limitations. First, only outpatients were surveyed; the inclusion of hospitalized patients is an important next step in assessing the country’s health literacy levels. Nonetheless, the importance of understanding the health literacy of the large and growing ambulatory patient population cannot be overstated. Also, bias could have been introduced because of missing respondents. The large sample size and relatively high response rates are reassuring that our findings accurately represent health literacy levels in the country. Further, the cross-sectional methodology can assess correlation, but cannot determine causality. Finally, inherent to any survey of complex issues, such as health literacy, are influencing factors that may not have been fully addressed in this study, such as income and socioeconomic status.

## Conclusion

Healthcare is a rapidly expanding and innovating field in the UAE. The high proportions of patients with limited or inadequate health literacy in our study imply that the health literacy deficit is a public health challenge in the UAE. Understanding and improving health literacy is a critical step in optimizing healthcare utilization and improving individual and population health outcomes. Future studies should include the design and implementation of interventions to improve clinician recognition of individuals with inadequate health literacy, particularly in the older population, and assess the use of communication techniques appropriate for patients with low health literacy. Also, the uptake and success of community-based programs to improve health literacy is an important area for future research.

## Supporting information

S1 Checklist STROBE statement—checklist of items that should be included in reports of *cross-sectional studies*. (DOCX)

## Abbreviations

GCC: Gulf Cooperation Council
UAE: United Arab Emirates
